# 

**Published:** 1905-06-24

**Authors:** 


### NOTICE TO CORRESPONDENTS.

All MSS., Letters, Books for Review, and other matters intended for the Editor should be addressed The Editor, "The Hospital," The Hospital Buildings, 28 & 29 Southampton Street, Strand, W.C.

The Editor cannot undertake to return rejected MSS., even when accompanied by stamped directed envelope.

### Notice to Advertisers and Subscribers.

All Advertisements, Orders for Copies of The Hospital, and Business Communications should be addressed to **THE MANAGER (Not to the Editor),** The Hospital, 28 & 29 Southampton Street, Strand, London, W.C.

The Cost of Subscription, post free, is at follows :—Per week, 3½d. ; per uarter, 3s. 3d. ; per half-year, 6s. 6d. ; per annum, 13s. Foreign and olonial :—Per annum, 19s. 6d., payable, in all cases, in advance.

**NOTICE.—Vol. XXXVII. of "The Hospital" October 1904 to March 1905), in handsome cloth binding, will shortly be on sale at the office of this Journal, price 10s. 6d. Cases for binding the Numbers contained in this Volume 2s. Index to Vol. XXXVII., 3½d., post free.**

**The Monthly Part for June is now ready. Price 1s., by post, 1s. 3d.**

### Medical Appointments.

**Thomas Aubrey**, M.B.Lond., L.R.C.P.Lond., M.R.C.S. *pro tem.* Medical Officer for the Bitton District of the Warmley (Gloucestershire) Union. **William Brander**, M.B., Ch.B.Aberd., Resident Workhouse Medical Officer and Medical Officer for the Children's Homes, Middlesbrough Union. **James Montague Braund**, M.R.C.S., L.S.A., reappointed Medical Officer of Health for the Stratton (Cornwall) Rural District Council. **D. V. Cow**, B.A., B.C.Cantab., Clinical Assistant to the Chelsea Hospital for Women. **Reginald Anderson Earle**, L.S.A.Lond., Medical Officer and Public Vaccinator for the Weston-super-Mare District of the Axbridge Union. **Oliver Eaton**, L.R.C.P.Lond., M.R.C.S., D.P.H.Cantab., reappointed Medical Officer of Health of Exmouth, Devon. **J. B. G. Gidley-Moore**, L.R.C.P.Lond., M.R.C.P.Edin., Certifying Surgeon under the Factory and Workshop Act for the Ongar District of the county of Essex. **W. Hassard**, M.B.Dub., House Surgeon to the Weston-super-Mare Hospital. Captain **J. H. Horton**, I.M.S., Clinical Assistant to the Chelsea Hospital for Women. **A. Beresford Kingsford**, M.R.C.S., L.R.C.P.Eng., D.P.H.Cantab., Second Assistant Anæsthetist at University College Hospital. **W. S. Myles**, B.A., M.B., B.Ch.Dub., District Medical Officer and Public Vaccinator, Moora, West Australia. **S. Charan Roy**, M.B., C.M.Edin., Resident Medical Officer to the Leicester Poor Law Infirmary. **C. R. Salisbury**, M.R.C.S., L.R.C.P.Lond., D.P.H.Lond., Anæsthetist to the Metropolitan Hospital.

### The Nurse's Library

**How to Become a Nurse: The Nursing Profession, How and Where to Train.**

Edited by Sir Henry Burdett, K.C.B. New Edition, 2/- net; 2/4 post free.

**Elementary Treatise on the Light Treatment for Nurses.**

By James H. Sequeira, M.D. Lond. Crown 8vo., illustrated, cloth, 2/6 net; 2/9 post free.

**Elementary Anatomy and Surgery for Nurses.**

By William McAdam Eccles, M.S.Lond., M.B., F.R.C.S., L.R.C.P. Crown 8vo., profusely illustrated, cloth, 2/6 post free.

**Surgical Ward-Work and Nursing.**

By Alexander Miles, M.D. Edin., C.M., F.R.C.S.E. Second Edition, enlarged and entirely revised. Demy 8vo. copiously illustrated, cloth gilt, 3/6 net, 3/10 post free.

**The Nurse's Pronouncing Dictionary of Medical Terms and Nursing Treatment.**

Compiled by Honnor Morten. Fifth Edition. Revised, with Phonetic Pronunciations, by Mary I. Burdett. Demy 16mo. (suitable for the apron pocket), bound in cloth boards, 160 pp., 2/- post free. In handsome leather, gilt, 2/6 net; 2/8 post free.

**Ophthalmic Nursing.**

By Sydney Stephenson, M.B., F.R.C.S.E. New and Revised Edition. Crown 8vo., profusely illustrated with Original Drawings, list of Instruments required, and a Glossary of Terms. Cloth, 3/6 net; 3/10 post free.

**A Complete Handbook of Midwifery.**

By J. K. Watson, M.D. Edin., Author of "A Handbook for Nurses." Crown 8vo., over 350 pp., profusely illustrated with 143 Diagrams. 6/- net; 6/4 post free.

**Hospital Sisters and their Duties.**

By Eva C. E. Luckes, Matron, London Hospital. Third Edition, enlarged and partly re-written. Crown 8vo., about 200 pp., cloth gilt, 2/6 post free.

**The Examination of the Urine.**

By J. K. Watson, M.D., M.B., C.M., Author of "A Handbook for Nurses." Demy 16mo., cloth boards, 1/- net post free.

**The Scientific Press, Ltd.**

Publishers of Nursing Text-Books, Case-Books, Charts, &c., of all descriptions.

***WRITE FOR NEW CATALOGUE.***

28 & 29 SOUTHAMPTON STREET, STRAND, LONDON, W.C.

### The NURSING MIRROR.

**The Nurse's Own Paper.**

**Price ONE PENNY Weekly.**

Of all Booksellers and Newsagents, or posted direct from the Office.

*Write for Subscription Rates to The Manager,*

THE NURSING MIRROR, The Hospital Building, 28 & 29 Southampton Street, Strand, London, W.C.

